# Predicting sustainable food consumption across borders based on the theory of planned behavior: A meta-analytic structural equation model

**DOI:** 10.1371/journal.pone.0275312

**Published:** 2022-11-16

**Authors:** Xin Shen, Qianhui Xu, Qiao Liu

**Affiliations:** College of Economics and Management, Shanghai Ocean University, Shanghai, China; Szechenyi Istvan University: Szechenyi Istvan Egyetem, HUNGARY

## Abstract

Interest in sustainable food consumption has gradually increased over the previous third decades. Despite substantial studies addressing various topics connected to sustainable food consumption, little research systematically evaluates which factors influence consumers’ purchase of sustainable food. We aim to integrate preliminary findings, compare four original and extended models of the theory of planned behavior (TPB) in the context of sustainable food consumption, and identify measurement and situational moderators using a meta-analytic structural equation modeling approach. The results show that attitude (ATT), subjective norms (SN), and perceived behavioral control (PBC) were most strongly positively correlated with a purchase intention (PI) of sustainable food. Furthermore, the analysis of the moderating effects revealed significant differences in the relationship between PBC and purchase behavior (PB) and between SN and PI in developing and developed countries. In addition, by comparing four original and extended TPB models, this study proposes a theoretical framework to affect customers’ PI of sustainable food. The findings of this study can be used as a foundation for company marketing and government environmental protection promotion.

## Introduction

Humanity confronts various environmental concerns, such as global warming, resource depletion, and the accelerating extinction of species. To overcome these concerns, international and interdisciplinary efforts must identify the key drivers and processes underlying the behavior causing these challenges, forecast their evolution over time, and ultimately change the system enough to mitigate negative consequences. The United Nations, in particular, has identified sustainable consumption as one of its primary objectives for achieving environmental sustainability [1]. Food sustainability is one of the critical strategies for attaining ecological sustainability and has been on the policy agenda in many nations [2, 3].

Technological advancement and government regulation play a significant role in food sustainability. However, the contribution of individuals’ behavior should not be overlooked. Consumers are paying more and more attention to the environment, which directly impacts the changes in individual lifestyles and values. As consumers become increasingly concerned about environmental, nutritional, and health-related problems, the demand for sustainable foods and beverages is rising [4]. This rising popularity has motivated researchers to delve deeper into the motivational drivers of sustainable product consumption. The profiles of consumers of sustainable products in the existing research primarily comprise the personal determinants driving sustainable purchases, willingness to pay for sustainable products, and the main hurdles to acquiring sustainable products [5–9].

Despite the considerable evidence, the determinants of sustainable purchasing behavior remain uncertain, as studies have reached different conclusions based on different scenarios. The theory of planned behavior (TPB) [10] is a robust theoretical model for predicting intention and behavior. In the context of food fields, the TPB was widely used to reveal how motivational factors determine food choices, such as organic foods and sustainable foods. However, although the TPB is traditional in terms of sustainable food, the understanding among scholars of the prediction of sustainable food consumption is not uniform. Many studies show that attitude (ATT), subjective norms (SN), and perceived behavioral control (PBC) have a significant and positive role in predicting sustainable food purchase intention (PI) and purchase behavior (PB). However, previous research into organic consumption has questioned the role of SN in the formation of individuals’ attitudes, along with the relation between PBC and ATT, which is not significant [11]. Notably, some recent studies on the role of SN to PI show completely different results. Dangi et al. [12] and Lin et at. [13] find that ATT and PBC have a significant positive impact on the intention to buy organic food. At the same time, SN is weak and barely powerful to meaning when conducted in an emerging economy. In another study of an emerging economy, Nguyen et al. [14] argue that ATT, SN, and PBC toward organic food purchase were positively related to PI. Testa et al. [2] suggest that PI positively influences purchasing behavior to buy organic food products and negatively by SN. So far, it is not entirely clear which are direct determinants of behavior or those that have a mediated influence. Thus, a synthesis analysis of the scenarios that impact the model is needed.

This study aims to integrate preliminary findings, compare four original and extended models of the TPB in the context of sustainable food consumption, and identify measurement and situational moderators using a meta-analytic structural equation modelling approach [15].

Specifically, we explore the relationship between ATT, SN, PBC, and PI and the effect of behavioral intention on actual behavior based on 42 previous empirical studies’ effect size of the TPB model of sustainable food consumption. Therefore, the first research question proposed in this paper is: in the empirical research of planned behavior theory on sustainable food consumption, how strong is the relationship between ATT, SN, PBC, and PI, and how strong is the effect of PI on actual behavior?

Second, we advance the existing meta-analyses [16–18] by studying country-level moderators that can impact the relationship between sustainable food consumption behavior determinants and their consequences on the TPB model. By reviewing the literature, we propose two possible moderators, including the degree of national economic development and national culture, to explain variations in studies and inconsistencies in the correlations between TPB dimensions. First and foremost, the study of sustainable food consumption behavior will benefit from a cross-country/cultural perspective due to global interest in environmental behavior, public health, climate change, and the need to pursue global-scale, cross-country solutions. In addition, the direction and strength of the correlation between TPB antecedents, behavioral intention, and actual conduct have varied in previous empirical studies, suggesting that moderating variables may exist. Understanding these moderators is crucial for providing a guide for food producers, marketers, and policymakers when making decisions about food products’ sustainability. This leads to the second research question: how do the two variables of national economic development and national culture adjust the above relationship?

Third, we intend to go beyond the classic framework of the TPB by analyzing a series of relationships rarely studied in the literature on sustainable consumer behavior and comparing different models from the original TPB to explore other mediating effects: 1) SN-ATT-PI-PB; 2) SN-PBC-PI-PB; 3) SN-PBC-PB; 4) SN-PI-PB.

The remainder of this paper is organized as follows: Section 2 outlines the conceptual model developed for the present study and includes a literature review; Section 3 explains the research methodology, which provides for the concept of meta-analysis, selection criteria related to the empirical studies, and how the data collected for the analysis; Section 4 describes the empirical results of overall estimates, moderators analysis, a test of models and mediating analysis; Section 5 discusses of results and implications; Section 6 provides conclusions and includes research limitations and suggestions for future work.

## Theoretical backgrounds

### The theory of planned behavior concerning sustainable food consumption

The TPB has been used successfully in food selection [19–22].

#### Attitude

Attitude is a psychological construct that conveys an individual’s global positive/negative assessment of a particular behavior; the more positive the attitude, the stronger the intention to express that behavior [19]. Some studies [23, 24] have proven that customers’ decisions are significantly influenced by their positive (or negative) attitudes regarding these items and their current alternatives, based on a complex combination of beliefs, motives, and experiences. Sparks and Shepherd [20] argue that attitude appears to be critical in determining behavior by directly affecting the intention of buying organic vegetables. However, there are some different opinions about an attitude towards consumption. Other studies [23, 25] have found that a positive attitude toward organic food is insufficient to stimulate their purchase since most customers are unwilling to pay a hefty premium price for them and consider other factors, such as limited availability in retail outlets. In addition, the strength of the relationship between attitude and behavioral intention in the case of sustainable food consumption differs significantly among research. For instance, a recent study by Boobalan [22] shows a significant association (r = 0.771) between a sample of individuals from India and the United States, whilst AI Mamun [26] finds a more moderate one (r = 0.504) in a Malaysian selection. Ahmed [27] found a minor connection (r = 0.238). As a result, while most research that used the TPB to study the intention to purchase and consume sustainable food indicated the critical role of attitude in influencing buying intention, the strength of this association remains unclear.

#### Subjective norm

Subjective norms are associated with the social influence or pressure on people to engage or refrain from a specific action [10]. The TPB mainly focuses on the role of injunctive norms. In particular, subjective norms express normative influence related to what the most critical referent individuals consider acceptable or unacceptable behavior [28]. Previous study findings indicate an inconclusive association between subjective norms and purchase intention or behavior pairs. According to Li et al. [9], China has a significant association between subjective norms and the desire to consume ecologically friendly farmed products. According to specific research, SN has a minor impact on organic or green food consumption [7, 13, 29]. Sparks and Shepherd [20] stress subjective norms in their investigation, although their explanatory power was relatively modest and substantial. However, the significance of personal criteria in influencing intentions has been neglected in many earlier research studies on organic food purchasing behavior [1]. Armitage and Conner [19] claim that the normative component of TPB may be the weakest of the model’s features. More astonishingly, Magnusson et al. [30] eliminate subjective norms from the suggested model in their research of organic foods in Sweden. Therefore, the initial study demonstrates that the correlation between personal criteria and intention has not been agreed upon.

#### Perceived behavioral control

Perceived behavioral control refers to an individual’s impression of the elements that may promote or obstruct the manifestation of behavior [31]. According to Ajzen’s model [10], PBC affects actual behavior only when it is not entirely under the individual’s volitional control. Typically, purchasing organic food hurdles are related to this type of product’s higher pricing and scarcity [31]. The impact of PBC on purchasing intention differs among research. For example, Li et al. [9] discover an r = 0.809 connection, but Yazdanpanah and Forouzani [32] find a non-significant correlation. As a result, several questions about the impact of PBC on organic food purchasing intentions remain unanswered.

### Moderate variable

Scholars’ research on the relationship between ATT, SN, and PBC and sustainable food purchase intention and actual consumption is limited by sample space. They cannot comprehensively and deeply explore the influence of potential regulatory variables, resulting in inconsistent research conclusions. With the help of a meta-analysis method, this study examines the relationship between ATT, SN, and PBC and sustainable food purchase intention and actual consumption based on a large sample across space and time. The study discusses the potential moderating effects of relevant variables, including measurement factors (sampling area) and situational factors (national culture).

#### Sample area

There are differences in consumption views of people in different countries or regions with different levels of economic development. Consumers in today’s world are increasingly concerned and aware of consuming eco-friendly food. According to previous research, sustainable food is healthier, more nutritious, and tastes better [33, 34]. As a result, purchasers have formed a positive attitude towards sustainable food consumption. Sustainable food farming and buying have proliferated in developing countries in recent years. However, buying sustainable food was ultimately the mindset of consumers in developed countries in the early days. Furthermore, as sustainable food prices are generally higher than ordinary non-sustainable food, and people in developed countries have more purchasing power than people in developing countries, we believe that developed countries will be more inclined to consume sustainable food than developing countries.

#### National culture

Attitudes, subjective norms, perceived behavioral control, and the relationship between behavioral intention and actual behavior may differ across cultural contexts, resulting in inconsistent relationships across study variables. Hofstede [35] proposed the uncertainty avoidance index (UAI)as one of the significant evaluation indexes for defining national culture. The degree to which a society tolerates uncertainty and ambiguity is defined as uncertainty avoidance. It also manifests as tension and the desire for predictability: the desire for written and unwritten rules. It assesses how a society deals with strange, unexpected events and change pressures. Controlling the evolution of these events is a strategy to minimize uncertainty and risk-taking, and this cultural dimension informs the philosophy of control. Societies with a low level of uncertainty avoidance are more open to change, less focused on control, have fewer rules and regulations, and have more freedom in their instructions. On the other hand, cultures with a high degree of uncertainty avoidance are less tolerant of change and tend to decrease dread of the unknown by using strong norms, regulations, and laws as control mechanisms. According to Hofstede [35], Uncertainty avoidance is intimately tied to formalization. Thus, consuming sustainable food is a way for people in nations with high uncertainty avoidance to regulate their health and reduce uncertainty. Ultimately, eating sustainable, green, and sustainably consumed foods in these cultural contexts reduces the likelihood of uncertain outcomes such as poor health and living environment. Therefore, we expect that intolerance of the unknown differentiates societies with high uncertainty avoidance and makes people more inclined to eat sustainable food.

## Methodology

### Sample collection and variable coding

The Web of Science and SCOPUS databases were searched for this study. The following terms and combinations are used as research keywords for titles, keywords, and abstracts: (“organic food” OR “green food” OR “natural” OR “Eco-friendly” OR “purchas*” OR “recycled” OR “nontoxic” OR “eating” OR “sustainable”) AND (“theory of planned behav*” OR “planned behav*” OR “Ajzen”). The results are extracted from online search engines and recorded in a comprehensive database. Studies with duplicate entries or basic missing information were excluded. A total of 5,223 studies were retrieved due to the wide range of keywords used. To avoid omission, many relevant studies are covered, and references to reviews and related articles are manually searched. The literature retrieval of this study will be completed on the 31st of July 2021.

In the retrieval process of articles, the literature is first screened to check whether the title and abstract meet the specific requirements. A total of 453 initial articles are obtained after initial screening. Based on the concept of the meta-analysis method and the principle of the structural equation model, the criteria for inclusion in the MASEM study are (1) empirical papers using survey data or secondary data. (2) All studies adopt a quantitative approach to Ajzen’s theoretical model of planned behavior and follow Ajzen’s original operational definition [10, 36]. (3) All studies need to assess willingness to buy or consume organic or sustainably produced food. (4) To calculate the effect size, each study must report at least one pair of Pearson correlation coefficients, such as attitude—intention, subjective norm—intention, and perceived behavioral control–intention. (5) The samples between studies should be independent. If two studies have duplicate samples, more detailed studies should be selected. (6) Subjects were adults over 18 years old. (7) The paper informs the sample size. (8) Must be published in English in a peer-reviewed academic journal.

**Fig 1:**
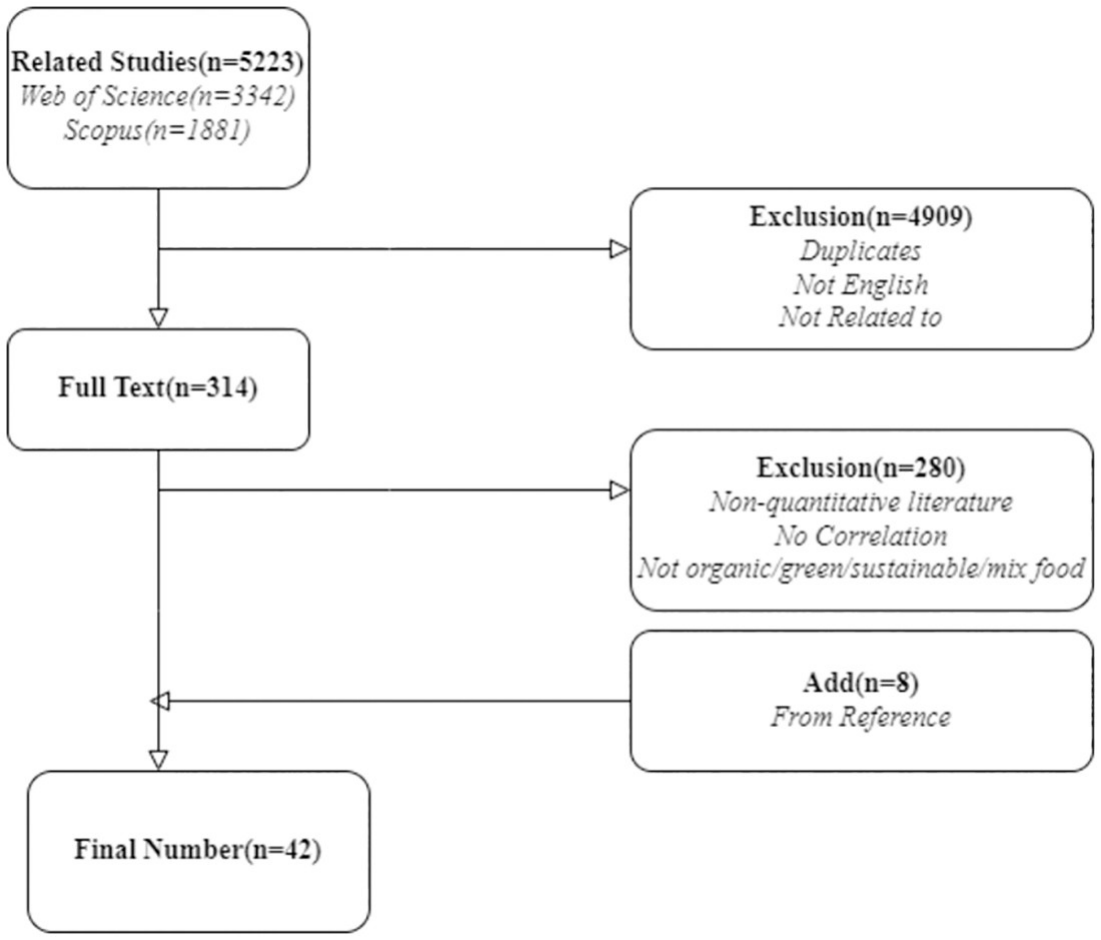
Literature screening process.

After screening according to the eligibility above criteria, the final database for meta-analysis comprised 42 publications, providing 50 different studies with 23,947 participants. Fig 1 sketches the process of literature selection and exclusion. Surprisingly, only 12 of the 50 studies revealed correlations between actual behavior and other TPB structures, even though these 12 studies represented approximately a third of the sample (6,169 participants).

All authors agreed on the definition of each dimension/variable before coding the literature. Authors coded independently and discussed and solved the ambiguity generated in the coding process. Specifically, the documents included in the meta-analysis are coded as follows: Literature information (author name and literature publication time), sample size, output results, correlation statistics (Pearson’s correlation coefficient r between variables or other statistics that can be converted into correlation coefficient and Cronbach’s alpha), sampling region, national culture, data source, etc. The correlation coefficient r of the effect size in the literature is coded as an effect size value for each article as an independent sample. A complete list of the studies considered in the current review and the related classifications based on the above variables, is available in Table 1. The data extracted from the study are shown in Table 2.

**Table 1:** Summary of the studies considered for the meta-analysis.

Author(s), year	Sample size	Food type	Sample country	Uncertainty Avoidance	Journal name
Al-Swidi et al., 2014 [21]	184	Organic food	Pakistan	70	British Food Journal
Arvola et al.1, 2008 [59]	202	Organic apples	Italy	75	Appetite
Arvola et al.2, 2008 [59]	270	Organic apples	Finland	59	Appetite
Arvola et al.3, 2008 [59]	200	Organic apples	UK	35	Appetite
Arvola et al.4, 2008 [59]	202	Organic pizza	Italy	75	Appetite
Arvola et al.5, 2008 [59]	270	Organic pizza	Finland	59	Appetite
Arvola et al.6, 2008 [59]	193	Organic pizza	UK	35	Appetite
Bamberg, 2002 [60]	320	Organic food	Germany	65	Journal of Economic Psychology
Dean, Raats, & Shepherd, 2012 [61]	501	Organic tomato sauce	UK	35	Journal of Applied Social Psychology
Dean et al., 2012 [61]	499	Organic tomatoes	UK	35	Journal of Applied Social Psychology
Dowd et al., 2013 [62]	137	Sustainably sourced food	Australia	51	Appetite
Guido et al., 2009 [31]	207	Organic food	Italy&France	/	International Economic Review
Honkanen et al., 2015 [63]	755	Sustainable seafood	UK	35	British Food Journal
Lee et al.,2015 [64]	482	Organic coffee	South Korea	85	International Journal of Contemporary Hospitality Management
Lodorfos et al.,2008 [23]	144	Organic food	UK	35	Journal of Food Products Marketing
Onwezen et al., 2014 [65]	944	Organic food	Netherlands	53	European Journal of Social Psychology
Robinson et al.,2002 [66]	547	Organic food	US	46	Journal of Nutrition Education and Behavior
Sparks et al.,1992 [20]	261	Organically produced vegetables	UK	35	Social Psychology Quarterly
Vermeir et al.,2008 [67]	456	Sustainable dairy	Belgium	94	Ecological Economics
Yadav et al.,2016 [1]	220	Organic food	India	40	Appetite
Yazdanpanah et al.,2015 [32]	389	Organic food	Iran	59	Journal of Cleaner Production
Zagata,2012 [68]	1054	Organic food	Czech Republic	74	Appetite
Saleki et al.,2019 [69]	246	Organic fruit and vegetables	Malaysia	36	Journal of Agribusiness in Developing and Emerging Economies
Ryan et al.,2018 [70]	306	High Brand organic breakfast cereals	US	46	Journal of Retailing and Consumer Services
Ryan,2018 [70]	311	Low Brand organic breakfast cereals	US	46	Journal of Retailing and Consumer Services
Tewari,2021 [5]	348	Organic food	India	40	British Food Journal
Dangi et al.,2020 [12]	306	Organic food	India	40	Journal of Asia Business Studies
Nguyen et al.,2019 [14]	572	Organic food	Vietnam	30	Sustainability
Chu, 2018 [52]	1421	Organic food	China	30	Sustainability
Canova et al.1,2020 [3]	237	Organic food	Italy	75	Frontiers in Psychology
Canova et al.2,2020 [3]	227	Fresh organic fruit and vegetables	Italy	75	Frontiers in Psychology
Lin et al.,2021 [13]	300	Organic food	China	30	International Journal of Environmental Research and Public Health
Qi et al,2019 [71]	300	Green food	China	30	Appetite
Mamun et al.,2018 [26]	380	Green food	Malaysia	36	Journal of Environmental Management
Testa et al.,2019 [2]	79	Organic food	Italy	75	Business Strategy and the Environment
Boobalan et al.,2021 [72]	911	Organic food	India&US	/	Food Quality and Preference
Carfora et al.,2019 [73]	1509	Organic milk	Italy	75	Food Quality and Preference
Leyva-Hernández et al.,2021 [74]	204	Organic food	Mexico	82	Foods
Li et al.1, 2020 [53]	310	Organic food	China	30	Frontiers of Business Research in China
Shen et al.,2020 [75]	436	Vegetarian burgers	Taiwan	30	Foods
Li et al.2,2020 [9]	850	Environmentally friendly agricultural (EFA) food	China	30	Environmental Science and Pollution Research
Kareklas et al.,2014 [76]	302	Organic food	US	46	Journal of Advertising
ŽIBRET et al.,2018 [77]	601	Organic food	Slovenia	88	Teorija in Praksa
Qi et al,2021 [7]	360	Green food	China	30	Foods
Qi et al.,2021 [78]	1412	Green food	China	30	Food Quality and Preference
Carfora et al.,2021 [8]	1018	Natural food	Italy	75	Nutrients
Nagaraj,2021 [79]	438	Organic food	India	40	Journal of Retailing and Consumer Services
Ahmed et al.,2020 [27]	515	Organic food	China	30	Journal of Environmental Planning and Management
Boobalan et al.,2020 [22]	1370	Organic food	India&US	/	Journal of Retailing and Consumer Services
Farias et al.,2019 [29]	241	Organic fruit and vegetables	Brazil	76	Journal of Food Products Marketing

**Table 2:** Raw correlations considered for the meta-analytic procedures.

Author(s)+Year	Sample size (N)	ATT-SN	ATT-PBC	SN-PBC	ATT-PI	SN-PI	PBC-PI	ATT-PB	SN-PB	PBC-PB	PI-PB
Al-Swidi et al.,2014 [21]	184	0.562	0.18	0.314	0.798	0.696	0.216	n.a.	n.a.	n.a.	n.a.
Arvola et al.1, 2008 [59]	202	0.69	0.44	0.46	0.73	0.62	0.41	n.a.	n.a.	n.a.	n.a.
Arvola et al.2, 2008 [59]	270	0.52	0.22	0.28	0.6	0.56	0.31	n.a.	n.a.	n.a.	n.a.
Arvola et al.3, 2008 [59]	200	0.57	0.4	0.34	0.67	0.55	0.36	n.a.	n.a.	n.a.	n.a.
Arvola et al.4, 2008 [59]	202	0.76	0.35	0.36	0.71	0.64	0.24	n.a.	n.a.	n.a.	n.a.
Arvola et al.5, 2008 [59]	270	0.46	0.03	0.15	0.55	0.58	0.1	n.a.	n.a.	n.a.	n.a.
Arvola et al.6, 2008 [59]	193	0.51	0.26	0.21	0.51	0.38	0.16	n.a.	n.a.	n.a.	n.a.
Bamberg, 2002 [60]	320	0.41	0.45	0.32	0.48	0.4	0.55	0.48	0.17	0.31	0.34
Dean, Raats, & Shepherd, 2012 [61]	501	0.66	0.53	0.36	0.74	0.72	0.45	0.55	0.55	0.31	0.64
Dean et al., 2012 [61]	499	0.64	0.48	0.43	0.71	0.71	0.43	0.35	0.34	0.3	0.49
Dowd et al., 2013 [62]	137	0.44	0.3	0.3	0.68	0.55	0.51	n.a.	n.a.	n.a.	n.a.
Guido et al., 2009 [31]	207	0.04	0.11	0.22	0.27	0.46	0.42	n.a.	n.a.	n.a.	n.a.
Honkanen et al., 2015 [63]	755	0.317	0.228	0.13	0.574	0.561	0.319	n.a.	n.a.	n.a.	n.a.
Lee et al.,2015 [64]	482	0.266	0.183	0.136	0.303	0.491	0.27	n.a.	n.a.	n.a.	n.a.
Lodorfos et al.,2008 [23]	144	0.281	0.12	0.114	0.82	0.534	0.486	n.a.	n.a.	n.a.	n.a.
Onwezen et al., 2014 [65]	944	0.344	0.171	0.228	0.561	0.524	0.185	0.42	0.421	0.185	0.657
Robinson et al.,2002 [66]	547	0.476	0.259	0.299	0.459	0.382	0.332	n.a.	n.a.	n.a.	n.a.
Sparks et al.,1992 [20]	261	0.37	0.06	0.05	0.38	0.3	0.27	n.a.	n.a.	n.a.	n.a.
Vermeir et al.,2008 [67]	456	n.a.	n.a.	n.a.	0.666	0.371	0.389	n.a.	n.a.	n.a.	n.a.
Yadav et al.,2016 [1]	220	0.02	-0.03	-0.09	0.55	-0.02	0.15	n.a.	n.a.	n.a.	n.a.
Yazdanpanah et al.,2015 [32]	389	-0.025	0.003	0.075	0.65	0.049	-0.021	n.a.	n.a.	n.a.	n.a.
Zagata,2012 [68]	1054	0.391	0.388	0.222	0.518	0.497	0.388	0.239	0.272	0.204	0.338
Saleki et al.,2019 [69]	246	0.422	0.49	0.359	0.62	0.542	0.581	0.431	0.307	0.337	0.49
Ryan et al.,2018 [70]	306	n.a.	n.a.	n.a.	0.458	n.a.	n.a.	n.a.	n.a.	n.a.	n.a.
Ryan,2018 [70]	311	n.a.	n.a.	n.a.	0.485	n.a.	n.a.	n.a.	n.a.	n.a.	n.a.
Tewari,2021 [5]	348	n.a.	0.307	n.a.	0.469	n.a.	0.594	n.a.	n.a.	n.a.	n.a.
Dangi et al.,2020 [12]	306	-0.029	0.604	0.054	0.346	0.163	0.556	n.a.	n.a.	n.a.	n.a.
Nguyen et al.,2019 [14]	572	0.553	0.364	0.375	0.573	0.578	0.528	n.a.	n.a.	n.a.	n.a.
Chu, 2018 [52]	1421	0.447	n.a.	n.a.	0.568	0.547	n.a.	n.a.	n.a.	n.a.	n.a.
Canova et al.1,2020 [3]	237	0.63	0.42	0.4	0.71	0.67	0.53	0.49	0.52	0.44	0.64
Canova et al.2,2020 [3]	227	0.37	0.2	0.24	0.67	0.51	0.44	0.48	0.41	0.18	0.58
Lin et al.,2021 [13]	300	0.524	0.489	0.395	0.46	0.367	0.38	n.a.	n.a.	n.a.	n.a.
Qi et al,2019 [71]	300	0.57	0.405	0.397	0.723	0.466	0.385	n.a.	n.a.	n.a.	n.a.
Mamun et al.,2018 [26]	380	0.199	0.402	0.31	0.504	0.248	0.557	0.324	0.067	0.5	0.486
Testa et al.,2019 [2]	79	0.432	0.646	0.539	n.a.	n.a.	n.a.	n.a.	n.a.	n.a.	n.a.
Boobalan et al.,2021 [72]	911	0.61	0.45	0.4	0.67	0.65	0.5	n.a.	n.a.	n.a.	n.a.
Carfora et al.,2019 [73]	1509	0.44	0.5	0.68	0.5	0.66	0.73	n.a.	n.a.	n.a.	n.a.
Leyva-Hernández et al.,2021 [74]	204	n.a.	n.a.	n.a.	0.715	n.a.	n.a.	n.a.	n.a.	n.a.	n.a.
Li et al.1, 2020 [53]	310	0.2	0.57	0.18	0.51	0.15	0.7	0.16	0.13	0.28	0.34
Shen et al.,2020 [75]	436	0.67	0.56	0.36	0.37	0.32	0.36	n.a.	n.a.	n.a.	n.a.
Li et al.2,2020 [9]	850	0.673	0.661	0.807	0.662	0.835	0.809	0.601	0.771	0.698	0.732
Kareklas et al.,2014 [76]	302	n.a.	n.a.	n.a.	0.53	n.a.	n.a.	n.a.	n.a.	n.a.	n.a.
ŽIBRET et al.,2018 [77]	601	0.24	0.3	0.3	0.31	0.45	0.36	0.16	0.11	0.26	0.2
Qi et al,2021 [7]	360	0.478	0.376	0.67	0.594	0.55	0.55	n.a.	n.a.	n.a.	n.a.
Qi et al.,2021 [78]	1412	n.a.	0.612	n.a.	0.575	n.a.	0.647	n.a.	n.a.	n.a.	n.a.
Carfora et al.,2021 [8]	1018	0.48	0.63	0.73	0.65	0.4	0.54	n.a.	n.a.	n.a.	n.a.
Nagaraj,2021 [79]	438	n.a.	n.a.	n.a.	0.188	n.a.	n.a.	n.a.	n.a.	n.a.	n.a.
Ahmed et al.,2020 [27]	515	0.026	0.213	-0.014	0.238	0.192	0.495	n.a.	n.a.	n.a.	n.a.
Boobalan et al.,2020 [22]	1370	n.a.	0.536	n.a.	0.771	n.a.	0.468	n.a.	n.a.	n.a.	n.a.
Farias et al.,2019 [29]	241	0.217	n.a.	n.a.	0.692	0.326	n.a.	n.a.	n.a.	n.a.	n.a.

Notes: Raw correlations that were not reported by the original papers are marked with “n.a.”.

### MASEM analysis process

To test the strengths of the correlations between the components of the theory of planned behavior regarding the purchase and consumption of sustainable food products, a meta-analytical structural equation model (MASEM) was used as a complementary advantage of meta-analysis and the structural equation model. This was accomplished using meta-analytical approaches that pooled the multiple correlation matrices accessible in the research before analyzing the results with structural equation models.

#### Effect quantity calculation

The meta-analysis in this study is performed using the professional software Comprehensive Meta-Analysis (CMA) 3.0. The Pearson correlation coefficient was chosen as the effect size in this investigation. Cohen [37] developed a rule of thumb that categorizes correlation coefficients as small, medium, or big, with values of roughly 0.10, 0.30, and 0.50. A small effect size suggests that the variables are perhaps independent, a medium effect size indicates that the covariance is only partially true, and a big effect size means that the covariance between the variables under consideration is (nearly) perfect.

#### Model selection and heterogeneity test

The fixed-effects model or random-effects model is mainly used in meta-analysis. Fixed effects models assume that all studies share the actual effect size, while random effects models believe that different studies have different effect sizes. According to Field and Gillett [38], researchers should select a suitable model based on the analysis performed and the inferences required. The random-effects model, in particular, is more appropriate when various researchers work in diverse circumstances, allowing the effect size to fluctuate randomly [39–41]. The random-effects model was employed in this analysis since most of the selected studies were conducted separately, drawing various samples from distinct populations.

Then, the Heterogeneity test was performed to confirm the rationality of the random effect model selection. Heterogeneity test methods mainly include the I^2^ test and the Q test. Values above 75% of I^2^ are given high heterogeneity, while values below 25% are assumed to be low heterogeneity. The null hypothesis of the Q test assumes complete homogeneity [41]; we can conclude that these studies are heterogeneous if the p-value is less than 0.05. If there is heterogeneity in the study, the moderating effect should be analyzed. We investigate the moderating impacts of national economic status and culture at various levels by analyzing and monitoring the coded data.

#### Publication bias

While conducting a meta-analysis, there may be publication bias; that is, articles with significant research results are more likely to be published. As a result of publication bias, the published literature cannot represent the entire state of finished research on the subject in a systematic and comprehensive manner [42]. Publication bias can significantly reduce the reliability of meta-analysis results. Increasing the sample size can effectively reduce the influence of publication bias. The publication bias test is conducted during the meta-analysis. In this study, two methods, the funnel plot and the fail-safe N test [43], are used for further testing.

#### Analysis of structural equation models

After meta-analysis, the joint correlation coefficient matrix between variables is obtained, and metaSEM R package [41] is used to examine the strengths of the correlations among constructs of TPB in sustainable food consumption. We aim to put original and modified TPB models to the test.

**Fig 2:**
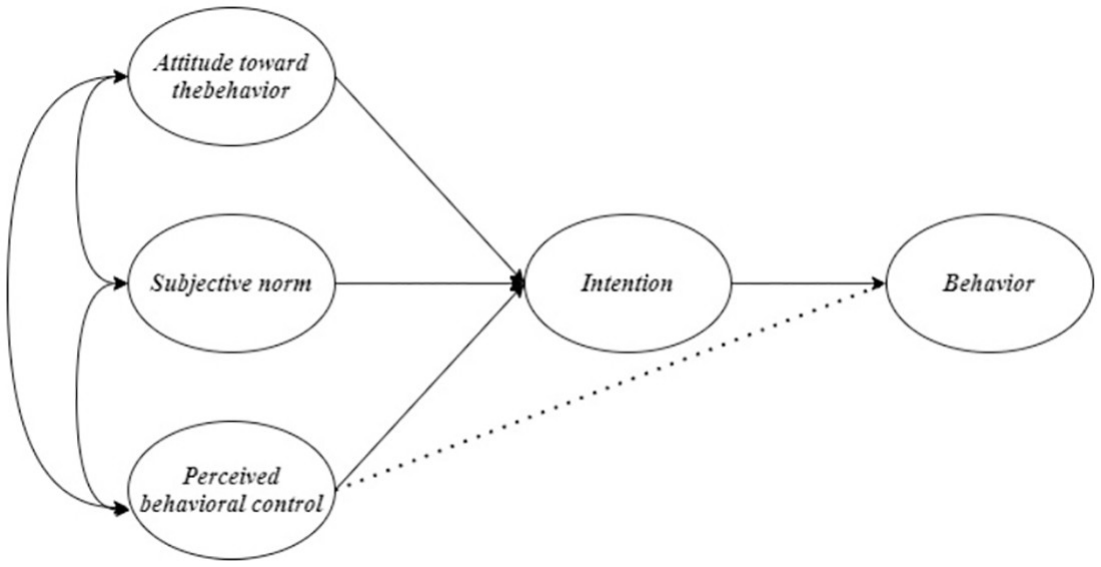
The original model was proposed in the theory of planned behavior [10] (Model A).

**Fig 3:**
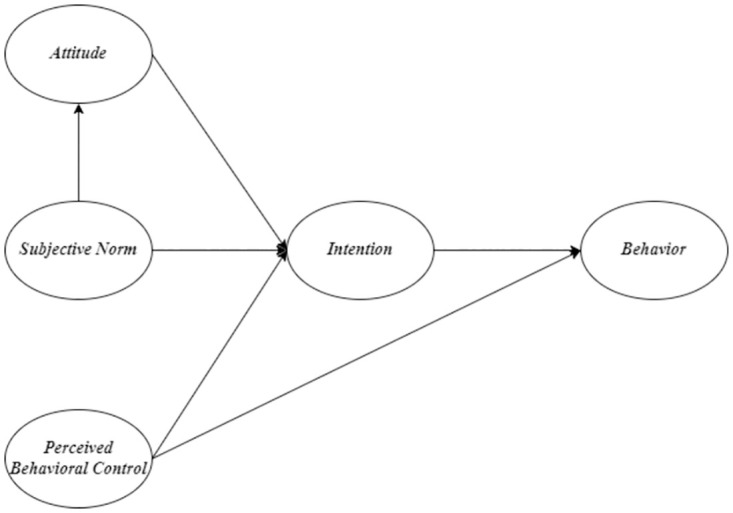
Model B.

**Fig 4:**
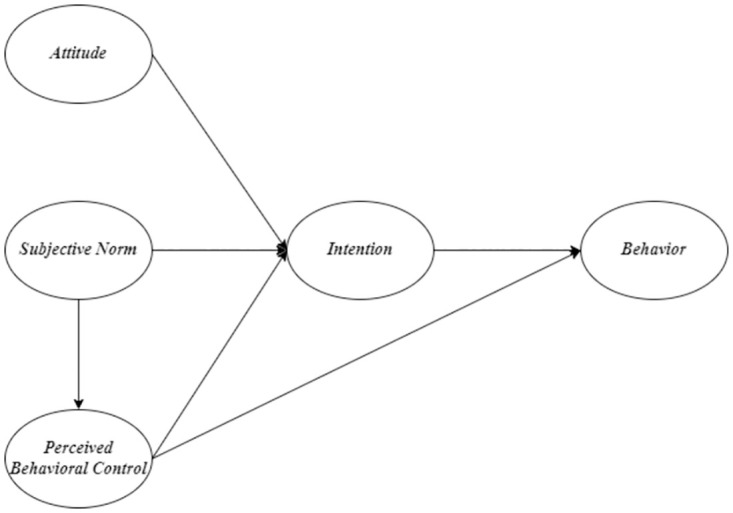
Model C.

**Fig 5:**
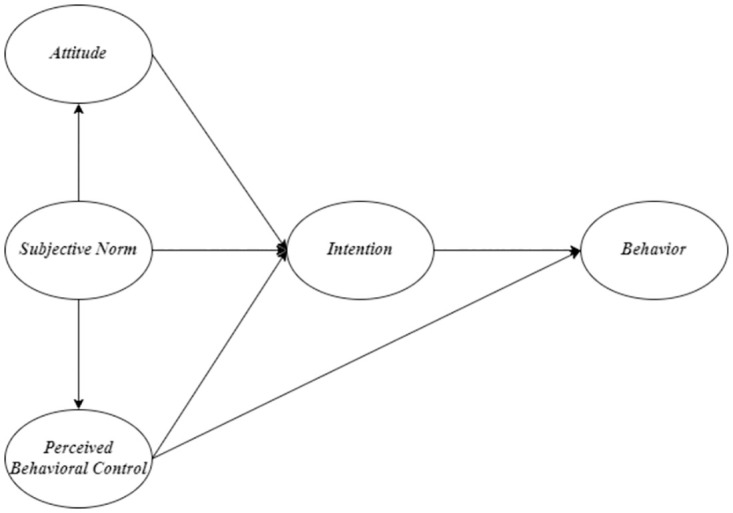
Model D.

Model A (Fig 2) is the original TPB proposed by Ajzen [10]. Model B (Fig 3), a modified TPB model, examines the additional direct effect of PBC on sustainable food consumption behavior proposed by Al-Swidi et al. [21] and Nguyen [14]. Model C (Fig 4) examines an additional direct effect of SN on PBC. Model D (Fig 5) is a hybrid of models B and C. The indicators commonly used to assess the goodness of a SEM are reported in the results section. In terms of markers of a satisfactory fit to the data, RMSEA≤0.05, CFI ≥0.90 (if not 0.95), SRMR≤0.08, and TLI≥ 0.90 are commonly considered.

## Results

### Main effects and heterogeneity test

Data are analyzed according to the above process, and the main effect is tested first. The results are shown in Table 3, which reports the number of effect size values, cumulative total sample size, mean effect size, 95% confidence interval, and Failsafe N of the relationship between ATT, SN, PBC, PI and PB.

**Table 3:** Main effect, heterogeneity test, and publication bias test research results.

	K	N	r	95%CI		Q-value	I^2^	Fail-safe N	5K+10
				LL	UL				
ATT-PB	12	6169	0.399***	0.277	0.508	190.735***	94.233	3117	70
ATT-PI	49	23868	0.578***	0.531	0.622	977.744***	95.091	5600	255
ATT-PBC	42	20268	0.369***	0.303	0.431	914.077***	95.515	3096	220
ATT-SN	41	18800	0.431***	0.368	0.491	948.986***	95.785	8412	215
PI-PB	12	6169	0.511***	0.402	0.606	344.821***	96.810	5785	70
PBC-PB	12	6169	0.345***	0.218	0.461	300.281***	96.337	2336	70
PBC-PI	42	20645	0.438***	0.376	0.496	1230.740***	96.669	8996	220
SN-PB	12	6169	0.362***	0.236	0.475	508.983***	97.839	2799	70
SN-PI	41	19177	0.489***	0.430	0.544	1147.314***	96.514	5614	215
SN-PBC	39	17138	0.330***	0.259	0.397	1539.366***	97.531	1341	205

Note: ATT: Attitude; SN: Subjective Norm; PBC: Perceived Behavior Control; PI: Purchase Intention; PB: Purchase Behavior; K: number of effect size N: sample size *** p<0.001; **p<0.01; * p<0.05; similar in the following tables.

First, the lower limit of the 95% confidence interval of the corresponding test results of each relational variable is greater than 0, indicating that these effect values have good reliability. The relationship between ATT, SN, PBC and PI is then investigated. The correlation coefficient between ATT and PI was the strongest (r = 0.578***). The correlation between SN and PI is slightly lower but still large (r = 0.489***). The correlation coefficient between PBC and the PI was the smallest (r = 0.438***). Similarly, there are similar results in examining the relationship between the antecedent variable of PI and PB. The correlation between ATT and PB was the strongest (r = 0.399***), followed by SN and PB (r = 0.362***), and finally, PBC and PB (r = 0.345***). In addition, there was a strong correlation between PI and PB (r = 0.511***).

Interestingly, the correlation between ATT, SN, and PBC also showed different magnitude. On the one hand, ATT (r = 0.369***) and SN (r = 0.330***) were equally strongly correlated with PBC. On the other hand, there was a moderate correlation between ATT and SN (r = 0.431***). The latter results appear to be of particular interest since, as shown in the following, they might suggest an indirect relationship between SN and PI mediated by ATT.

The heterogeneity test aims to test whether the effect sizes measured between studies are heterogeneous. As mentioned earlier, fixed-effects models are considered appropriate only when the differences between studies are minimal, and random-effects model tests should be used for a series of studies with high heterogeneity. Therefore, we use the random-effects model. We examined the I^2^ and Q test values: I^2^ values ranged from 94.23% to a maximum of 97.84% in the test correlation, thus indicating a very high overall heterogeneity between studies (see Table 3). Similarly, Q tests consistently report values associated with p values <0.001, thus confirming differences between studies.

### Publication bias test results

**Fig 6:**
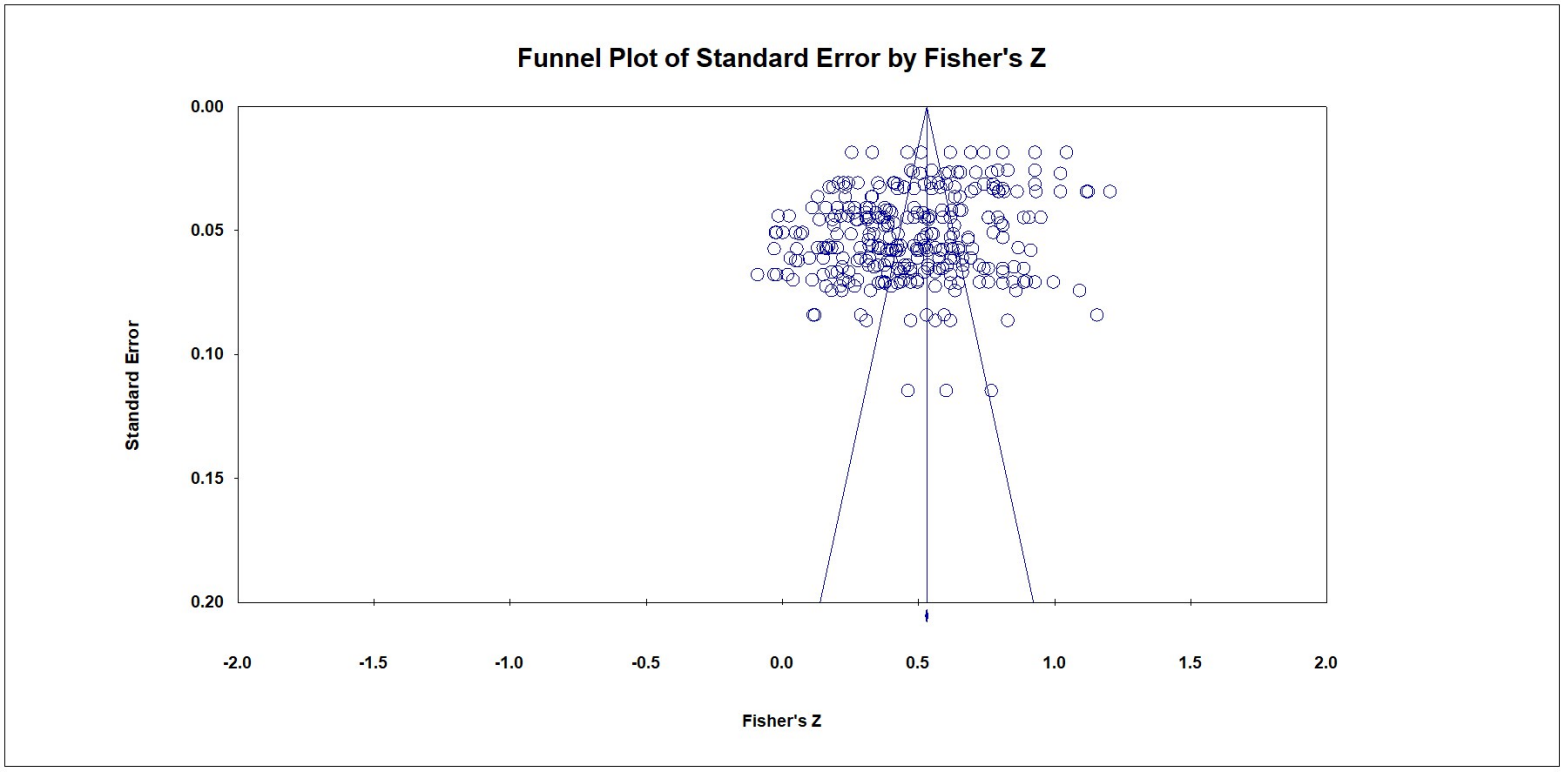
Literature screening process.

First, a funnel plot is utilized to examine the publication bias of the meta-analysis, as illustrated in Fig 6. The literature is dispersed on both sides of the total effect size, indicating no severe publication bias in the results. Failsafe N is used for the tests to exclude subjective factors, and the results are shown in Table 3. When the Failsafe N value is greater than 5K+10 (K represents the number of independent samples, that is, the number of effect size values), the larger the value, the more stable the analysis results are. The less likely the research conclusions are to be overturned. When the coefficient of the Fail-Safe N is less than 5K+10, it indicates the existence of publication bias [44]. The results in Table 3 show that the Failsafe N of SN and PBC is the smallest, with a value of 1341, which is greater than the corresponding critical value of 205 (i.e., 5*39+10), which means that in the case of a significance level of α = 0.05, an additional 1341 research papers are needed to deny the significant relationship between SN and PBC. The remaining Failsafe N values are much larger than the corresponding critical values. Therefore, there is no publication bias in this study.

### Moderator analysis

The heterogeneity test results show that the effect size values of all studies are highly heterogeneous. This study further tests the moderating effects of the sampling region and national culture on the relationship between variables (see Table 4).

**Table 4:** Results of moderator analysis.

Relationships	Moderator variables	Level	K	r	95%CI		Q-value
					LL	UL	
ATT-PB	Economic status	Developed country	7	0.432***	0.316	0.536	0.156
		Developing country	4	0.395***	0.233	0.535	
	Uncertainty Avoidance	Low	8	0.386***	0.265	0.496	0.138
		High	4	0.424***	0.252	0.570	
ATT-PI	Economic status	Developed country	26	0.588***	0.538	0.634	0.707
		Developing country	20	0.556***	0.496	0.611	
	Uncertainty Avoidance	Low	31	0.553***	0.499	0.602	3.537
		High	16	0.632***	0.565	0.690	
ATT-PBC	Economic status	Developed country	23	0.332***	0.246	0.413	1.373
		Developing country	16	0.407***	0.310	0.496	
	Uncertainty Avoidance	Low	26	0.388***	0.311	0.459	0.430
		High	14	0.345****	0.235	0.446	
ATT-SN	Economic status	Developed country	23	0.472***	0.393	0.543	2.645
		Developing country	16	0.369***	0.266	0.464	
	Uncertainty Avoidance	Low	24	0.420***	0.340	0.493	0.374
		High	15	0.458***	0.359	0.546	
PI-PB	Economic status	Developed country	7	0.537***	0.401	0.650	0.003
		Developing country	4	0.531***	0.344	0.678	
	Uncertainty Avoidance	Low	8	0.524***	0.387	0.638	0.119
		High	4	0.484***	0.275	0.650	
PBC-PB	Economic status	Developed country	7	0.276***	0.153	0.391	4.659**
		Developing country	4	0.478***	0.335	0.599	
	Uncertainty Avoidance	Low	8	0.373***	0.223	0.506	0.446
		High	4	0.286***	0.059	0.485	
PBC-PI	Economic status	Developed country	23	0.394***	0.300	0.481	2.615
		Developing country	16	0.495***	0.392	0.586	
	Uncertainty Avoidance	Low	26	0.464***	0.381	0.540	1.308
		High	14	0.382***	0.259	0.494	
SN-PB	Economic status	Developed country	7	0.390***	0.179	0.566	0.015
		Developing country	4	0.369***	0.082	0.600	
	Uncertainty Avoidance	Low	8	0.367***	0.163	0.541	0.011
		High	4	0.349***	0.051	0.590	
SN-PI	Economic status	Developed country	23	0.535***	0.455	0.606	3.743*
		Developing country	16	0.408***	0.298	0.508	
	Uncertainty Avoidance	Low	24	0.467***	0.381	0.544	0.483
		High	15	0.512***	0.408	0.603	
SN-PBC	Economic status	Developed country	23	0.327***	0.202	0.442	0.000
		Developing country	16	0.329***	0.168	0.473	
	Uncertainty Avoidance	Low	23	0.305***	0.183	0.418	0.456
		High	14	0.370***	0.218	0.504	

From the moderating effects of national economic development degree (developed vs. developing countries) on the relationship between ATT, SN, PBC, PI and PB, the relationship between PBC and PB is significantly different at the significance level α = 0.01 (Q = 4.659, P <0.01). Although there was a high positive correlation between PBC and PB in both developed and developing countries (0.276 (P <0.001) and 0.478 (P <0.001) respectively), the effect was more significant in developing countries. Besides, the relationship between SN and PI is significantly different at the significance level α = 0.01 (Q = 4.659, P <0.01). Although there was a high positive correlation between SN and PI in both developed and developing countries 0.535 (P <0.001) and 0.408 (P <0.001) respectively), the effect was more significant in developing countries. However, the moderating effects of other relationships were not significant.

Unfortunately, the moderating effects of national culture (high uncertainty avoidance vs. low uncertainty avoidance) on the relationship between ATT, SN, PBC, PI, and PB were insignificant for each connection.

### Test of models

Four TPB models, both original and modified, were assessed. Results and fit indexes are summarized in Table 5. Model A was assessed using 50 studies (sample size = 23,947), and the goodness-of-fit indexes were considerably beyond acceptable criteria (Model A: *χ*2 (2) = 5.988, p < 0.001; RMSEA = 0.009, SRMR = 0.000; TLI = 0.990; CFI = 0.998). These findings imply that TPB is very helpful in predicting sustainable food consumption behavior. R^2^ values for intention and behavior of sustainable food consumption were 0.572 and 0.687, respectively, indicating that the model accounts for approximately 57.2% of the explanation power in predicting intention and approximately 68.7% of the explanation power in predicting behavior of sustainable food consumption. The results suggest that ATT had the greatest impact on PI (b = 0.396, 95% CI = [0.334,0.455]), followed by SN (b = 0.252, 95% CI = [0.177,0.325]) and PBC (b = 0.196, 95% CI = [0.126,0.263]). The effect of PI on PB is very high (b = 0.483, 95% CI = [0.384,0.583]).

**Table 5:** Summary of the goodness-of-fit indices achieved for each tested MASEM.

Model	*χ*^2^(df)	p-value	RMSEA	95%LL	95%UL	SRMR	TLI	CFI	AIC	BIC
A	5.988 (2)	0.050	0.009	0.000	0.018	0.033	0.990	0.998	1.988	-14.179
B	57.790 (3)	0.000	0.028	0.022	0.034	0.071	0.904	0.971	51.790	27.539
C	58.421(3)	0.000	0.028	0.022	0.034	0.072	0.903	0.971	52.421	28.171
D	58.470(3)	0.000	0.028	0.022	0.034	0.072	0.903	0.971	52.497	28.219

Model B was an extension of model A that included the direct influence of SN on ATT. For the sample of 50 studies (sample size = 23,947), the indices reveal excellent fit: *χ*2 (3) = 57.790, p < 0.001; RMSEA = 0.028, SRMR = 0.071; TLI = 0.904; CFI = 0.971. R^2^ on intention and behavior of sustainable food consumption demonstrates a significant explanatory power on sustainable food consumption intention and behavior (54.5% and 69.3%, respectively). The order of strength of effect on intention for model B is different with that of model A, leading by attitude (b = 0.443, 95% CI = [0.387,0.501]), following by PBC (b = 0.266, 95% CI = [0.204,0.327]) and then SN (b = 0.150, 95% CI = [0.066,0.230]). The influence of intention on behavior of sustainable food consumption is the largest (b = 0.512, 95% CI = [0.418,0.590]), followed by the influence of PBC on sustainable food consumption behavior (b = 0.118, 95% CI = [0.024,0.219]).

Model C was a modified version of model A that had a direct link between SN and PBC. The goodness-of-fit indexes (model C: *χ*2 (3) = 58.421, p < 0.001; RMSEA = 0.028, SRMR = 0.072; TLI = 0.903; CFI = 0.971) were assessed using 50 studies (sample size = 23,947). R^2^ on intention and behavior of sustainable food consumption show the strong explanation power on intention and behavior of sustainable food consumption (56.2% and 67.8%, respectively). Similar with model B, the order of strength of effect first is attitude (b = 0.444, 95% CI = [0.381,0.506]), following by PBC (b = 0.267, 95% CI = [0.198,0.332]) and then SN (b = 0.149, 95% CI = [0.059,0.238]). The impact of PI on PB of sustainable food consumption is the strongest (b = 0.504, 95% CI = [0.411,0.598]), followed by the influence of PBC on PB of sustainable food consumption (b = 0.123, 95% CI = [0.015,0.228]).

Model D was a hybrid of models B and C. The goodness-of-fit indexes (model D: *χ*^2^ (3) = 58.470, p < 0.001; RMSEA = 0.028, SRMR = 0.072; TLI = 0.903; CFI = 0.971) were assessed using 50 studies (sample size = 23,947). R^2^ on intention and behavior of sustainable food consumption demonstrates a significant explanatory power on sustainable food consumption intention and behavior (56.3% and 67.9%, respectively). Similar with model B, the order of strength of effect first is attitude (b = 0.444, 95% CI = [0.380,0.507]), following by PBC (b = 0.266, 95% CI = [0.197,0.333]) and then SN (b = 0.150, 95% CI = [0.058,0.238]). The effect of PI on PB of sustainable food consumption is the strongest (b = 0.504, 95% CI = [0.410,0.598]), followed by the effect of PBC on PB of sustainable food consumption (b = 0.123, 95% CI = [0.014,0.229]).

### Mediating effects

For model D, we further do a mediation analysis and the results show in Table 6. We adopted bootstrapping to test the significance of the mediating effect. Firstly, from p-values less than 0.01 and confidence intervals of the effect of the bias-corrected percentile method and the percentile method not including 0 at 95% confidence interval, it can be concluded that all path results are positive and significant. Secondly, the results of the analysis from subjective norms to purchase behavior showed that SN-PI-PB had the largest value of 0.117, followed by SN-ATT-PI-PB with a value of 0.080, followed by SN-PBC-PB with a value of 0.055 and finally SN-PBC-PI-PB with a value of 0.029.

**Table 6:** Mediating effect test results.

Path	Point estimation	Bias-corrected 95%CI		Percentile 95%CI	
		LL	UL	LL	UL
SN-ATT-PI-PB	0.080**	0.076	0.084	0.076	0.084
SN-PBC-PI-PB	0.029**	0.027	0.031	0.027	0.031
SN-PBC-PB	0.055**	0.051	0.060	0.051	0.060
SN-PI-PB	0.117**	0.110	0.123	0.111	0.123

## Discussion

This study reviews the relationship between sustainable food’s ATT, SN, PBC, PI, and PB. Studies on samples from developed countries were first published in 1992, and a maximum of eight were published in 2008. Reflections on developing countries began to appear in 2014 and peaked in 2021. This reflects a growing global focus on sustainable food consumption. Consumers in developing countries are increasingly focusing on sustainable products due to improved quality of life and an emotional shift from conventional to healthy diets. Surprisingly, contributions on this topic may be found in various journals from other areas. This highlights the research’s multidisciplinary character and the broad interest of economists, dietitians, and social psychologists, to name a few. In addition, the samples of developed countries are mainly composed of the United States and Europe. In contrast, developing country samples are mostly comprised of growing economies like China and India. Their diversity underscores Peattie’s [45] perspective on green consumption’s international spread, which shows the globalization of environmental concerns.

The meta-analysis’ findings reveal the extent of individual relationships between TPB structures. Our results demonstrated that attitude has the most potent effect on intention of sustainable food consumption, followed by SN, while PBC had a minor influence on choice. These results suggest that individual ATT and SN have a major effect on the intention of sustainable food consumption, whereas PBC seems to have less influence. As one might expect, personal attitudes are the main factor influencing consumers’ willingness to buy. The correlation of this relationship (r = 0.578***) is near the reasonable limit of the predictive utility suggested by Ajzen [46]. Furthermore, the results also show that SN can significantly support the formation of PI in sustainable food. Contrary to popular belief that social norms may represent the weakest part of TPB [19], the main effect results reveal that the social sphere strongly influences the willingness to buy sustainable products in the context of a sustainable environment. Since consumer behavior is susceptible to the impact of other individuals and group regulations [47], relevant departments should enhance social norms of energy-saving and environmental preservation. The effect of PBC is primarily due to customers’ increased propensity to buy when they are more confident in their purchasing abilities, as demonstrated by earlier research [48, 49]. As a result, businesses should supply customers with trustworthy information on the benefits of sustainable products. Practical knowledge is critical for customers’ decision-making and can boost their purchase confidence. Likewise, if customers are aware of the environmental benefits of individual green purchasing behavior, their green buy intention will rise.

The study also shows that the relationship between PI and PB is stronger than the relationship between ATT, SN, PBC, and PB. On the one hand, this indicates that PI is the most significant predictor of PB. Still, it also supports the idea of the intention-behavior gap, which states that even the most potent intents may not convert into commensurate behaviors [50]. Few studies report a correlation between intention and actual action, influencing the reliability of the results. This problem, however, is frequent in research that does a meta-analysis of TPB in various settings. For example, in the study of Nguyen et al. [14] on knowledge sharing, only 11 of 26 studies revealed intention-behavior association. In another research by Schwenk and Möser [51] on environmental behavior, 11 studies out of 25 showed correlations between intention and behavior. Because often interrupted by investigation on the PI stage, it may be to buy sustainable food and affirmation of the consumer’s theory of planned behavior constitutes a threat to further, we strongly suggest that further research, in addition to considering other dimensions of the TPB, consider the participants in the purchase or consumption of sustainable food in terms of the actual behavior assessment. Measures of actual market behavior should be included in consumer behavior research. Since measuring potential customer behavior can be difficult because it requires observation of consumer behavior, we propose that at least two items be included in the questionnaire to investigate past consumer behavior.

Two additional moderators were explored to capture the roles of the sample area and national culture. Interestingly, the results confirmed the significant role of these dimensions. Specifically, the moderator role of the sample area dimension was found in the relationship between PBC and PB in consuming sustainable food. This study shows that developing countries have a much stronger effect of PBC on PB to consume sustainable food. This implies that increased education on the benefits of sustainable food in developing countries could increase the consumption of sustainable food by developing country residents. We also found the role of economic status in moderating the influence of SN on the intention to consume sustainable food. This means that SN is important for PI in both developed and developing countries which is in accordance with Chu [52]. And the results of this study show that developing countries have a much stronger effect of SN on PI to consume sustainable food. Sustainable foods in emerging countries like China and India have become famous for their health properties. In the past two decades, the sustainable food industry in China has experienced rapid development, among which, in 2020, the number of organic food units is 1,228 and the number of organic products is 4,466, increasing by 91.7% and 94.8%, respectively since 2003, according to China Green Food Development Center in 2021.

The final section of the analysis examines different TPB models and investigates the many correlations between their aspects using a MASEM. Among the four TPB models (Models A, B, C and D), our empirical results show that the original TPB seems to provide reasonable support in predicting PB of sustainable food with goodness-of-fit indices and strong explanation power on PI and PB of sustainable food. First, the finding related to the mediating effect of attitude toward sustainable foods on the relationship between SN and PI to purchase sustainable foods is statistically significant. This result is consistent with the empirical findings of Chu [52] and Farias et al. [29]. Furthermore, the results suggest that attitudes mediate between SN and PI, further influencing actual behavior. This result is in accordance with the empirical findings of Testa et al. [2], Li et al. [53] and Bamberg and Möser [54]. This implies that, if people who are meaningful to consumers offer opinions and positive attitudes toward sustainable foods, consumers will be more likely to have a positive intention to purchase sustainable foods. As Chang [55] suggested, there is a need for further investigation into the effect of social pressure on shaping attitudes. Beliefs about the expectations from the reference groups may affect the formation of individuals’ attitudes. This has been investigated by a few scholars [11, 21, 56] in the field of organic consumption. Our research has provided evidence of a significant causal path between subjective norms and attitudes leading towards behavior, arguing that attitudes towards buying sustainable food and subjective norms are not independent. Second, the finding related to the mediating effect of PBC on the relationship between SN and PI to purchase sustainable foods is statistically significant. This implies that, SN is directly associated with the degree of perceived behavioral control. People may use SN for judging how easy and beneficial the performance of a specific behavioral option would be. This result is consistent with the empirical findings of Bamberg and Möser [54]. When individuals perceive information fully and understand a situation, there may be a higher probability that sustainable food consumption occurs. Therefore, establishing a convenient and friendly environment to facilitate sustainable food consumption can be particularly important. Perceived behavioral control is directly influenced by subjective norms and intentional and actual behavior [57]. This result is worthy of further study: on the one hand, the relevant MASEM shows excellent fitting indicators; On the other hand, there is at least one point worth noting and deepening is that the correlation was observed only 12 times.

## Conclusion, limitations, and further research directions

Based on earlier research, this study uses a MASEM to examine the factors influencing consumers’ sustainable food purchase intentions. According to the theory of planned behavior, the elements influencing consumers’ sustainable buy intention fall into three constructs: ATT, SN, and PBC. The results show that ATT, SN, and PBC were most strongly positively correlated with a PI of sustainable food. The findings of this study add to the theoretical foundation for understanding consumers’ buy intentions for sustainable products and new methodologies and ideas for investigating the influencing elements of sustainable product purchasing intention. Furthermore, the analysis of the moderating effects revealed significant differences in the relationship between PBC and PB and between SN and PI in developing and developed countries, with the connection being more robust in developing countries. In addition, by comparing four original and expanded TPB models, this study proposes a theoretical framework to affect customers’ PI of sustainable food. The findings of this study can be used as a foundation for company marketing and government environmental protection promotion. Enterprise marketers may make greater use of the study’s findings to develop marketing strategies; government agencies can highlight the benefits of sustainable products and assist consumers in practicing environmental stewardship.

There are still some limitations in this study. First, because of the significant heterogeneity of the results, the accuracy of random effect size was reduced, and the small datasets added to the study limited our ability to investigate moderators that may assist the analysis. The second is in the process of literature collection; this paper chose between the quantity and quality of literature. Strict standards and high quality were selected to screen the literature to ensure the reliability of experimental data. As a result, some literature was not included in the meta-analysis. The third one is that this paper only explained the differences in existing research results from the sampling region and country culture due to the limited number of studies. There may still be other moderating variables that have an impact, which is worthy of further research.

We believe that at least three issues have emerged: first, the need for a more robust methodological exploration of the dimensions in future literature; second, the importance of determining whether the relationship between perceived behavioral control and actual behavior holds in the current context; and third, investigate whether the function of attitudes in mediating the relationship between subjective norms and behavioral intentions extends to other similar green items, such as locally produced food (or local specialities), fairtrade products, and even eco-friendly technological gadgets. Paul et al. [58] also confirmed the effectiveness of this recommendation for a variety of green products. Therefore, in addition to food, future studies should consider applying structural equation models to check the validity of the classic TPB model or the proposed extended TPB model for sustainable products.

## Supporting information

S1 Checklist PRISMA 2020 checklist. (PDF)
